# Nutritional Challenges in Duchenne Muscular Dystrophy

**DOI:** 10.3390/nu9060594

**Published:** 2017-06-10

**Authors:** Simona Salera, Francesca Menni, Maurizio Moggio, Sophie Guez, Monica Sciacco, Susanna Esposito

**Affiliations:** 1 Pediatric Highly Intensive Care Unit, Department of Pathophysiology and Transplantation, Università degli Studi di Milano, Fondazione IRCCS Ca’ Granda Ospedale Maggiore Policlinico, 20122 Milan, Italy; simona.salera@policlinico.mi.it (S.S.); francescamenni@hotmail.com (F.M.); sguez_2000@yahoo.com (S.G.); 2 Neuromuscular and Rare Disease Unit, Department of Neuroscience, Foundation IRCCS Ca’ Granda Ospedale Maggiore Policlinico, University of Milan, 20122 Milan, Italy; maurizio.moggio@unimi.it (M.M.); monica.sciacco@policlinico.mi.it (M.S.); 3 Pediatric Clinic, Università degli Studi di Perugia, 06129 Perugia, Italy

**Keywords:** dysphagia, hyponutrition, neuromuscular disease, nutrition, osteoporosis, overnutrition

## Abstract

Neuromuscular diseases (NMDs) represent a heterogeneous group of acquired or inherited conditions. Nutritional complications are frequent in NMDs, but they are sometimes underestimated. With the prolongation of survival in patients with NMDs, there are several nutritional aspects that are important to consider, including the deleterious effects of overnutrition on glucose metabolism, mobility, and respiratory and cardiologic functions; the impact of hyponutrition on muscle and ventilatory function; constipation and other gastrointestinal complications; chewing/swallowing difficulties with an increased risk of aspiration that predisposes to infectious diseases and respiratory complications; as well as osteoporosis with an associated increased risk of fractures. The aim of this review is to provide a comprehensive analysis of the nutritional aspects and complications that can start in children with Duchenne muscular dystrophy (DMD) and increase with ageing. These aspects should be considered in the transition from paediatric clinics to adult services. It is shown that appropriate nutritional care can help to improve the quality of life of DMD patients, and a multidisciplinary team is needed to support nutrition challenges in DMD patients. However, studies on the prevalence of overnutrition and undernutrition, gastrointestinal complications, infectious diseases, dysphagia, and reduced bone mass in the different types of NMDs are needed, and appropriate percentiles of weight, height, body mass index, and body composition appear to be extremely important to improve the management of patients with NMD.

## 1. Introduction

Neuromuscular diseases (NMDs) represent a heterogeneous group of acquired or inherited conditions. They can be classified into four major categories on the basis of their neuroanatomical localization: motor neuron diseases, neuropathies, disorders of the neuromuscular junction, and myopathies [1,2]. The predominant and common symptom across NMDs is hypotonia, which results in muscle weakness, with consequent fatigue, reduced mobility, and diminished physical work capacity. In addition, orthopaedic, cardiac, infectious, and respiratory problems are often present, which result in worsening quality of life for NMD patients and their families [1,2]. The prevention and appropriate treatment of infectious diseases as well as improvement in respiratory and cardiologic assistance, physiotherapy, and other aspects of treatment has recently changed the natural course of NMDs with an increased number of patients living into adulthood [3]. However, despite the improvement in survival, disease progression is only slowed with these therapeutic advances, leading to profound muscle weakness, disability, and complex medical and social needs that worsen with the ageing of patients [4]. Furthermore, positive medical achievements sometimes result in the appearance of treatment-related adverse events (i.e., chronic corticosteroid therapy may cause short stature, delayed puberty, obesity, osteoporosis, infections, and behavioural problems) [5]. At the same time, the appearance of adulthood-related problems (i.e., cardiovascular risk and metabolic syndrome) is a consequence of prolonged life [5].

Nutritional complications are often present in NMDs and worsen with age. Specifically, as the child grows up, there are several nutritional aspects of the disease that are important to consider. Among these aspects, there are the deleterious effects of overnutrition on glucose metabolism, mobility, and respiratory and cardiologic functions; the impact of hyponutrition on muscle and ventilatory function; constipation and other gastrointestinal complications; chewing/swallowing difficulties with an increased risk of aspiration that predisposes to infectious diseases and respiratory complications; as well as osteoporosis. Among these aspects are the deleterious effects of overnutrition, which are associated with an increased risk of fractures [6]. However, appropriate nutritional care can help to improve the quality of life of NMD patients and their families [6,7]. The aim of this review is to provide a comprehensive analysis of the nutritional aspects and complications that can start in children with NMD but increase with ageing. These aspects should be considered in the transition from cohesive paediatric clinics to disjointed adult services. Due to the limited availability of data on other NMDs, this manuscript focuses on Duchenne muscular dystrophy (DMD).

## 2. Anthropometrics and Growth Charts

Among NMDs, DMD is the only disease for which specific growth charts are available. At birth, weight and length in males with DMD are similar to standard distribution patterns, suggesting that the disease progression is potentially responsible for the differences in height and alterations in body weight [8]. Normal growth charts do not consider the progressive loss of muscle that occurs as muscular dystrophy progresses throughout childhood. Therefore, normal weight results on standard growth charts implies that fat tissue is accumulating [9].

In 1988, Griffiths and Edwards proposed a growth chart for boys with DMD that accounted for progressive muscle loss at a rate of 4% decline per year, thus providing ideal weight guidelines for weight control in the disease [9]. In 2013, West et al. presented a set of growth curves derived from a large cohort of male youths with DMD (i.e., 513 ambulatory males, 2–12 years old) [10]. These curves for weight, height, and body mass index (BMI) demonstrate that DMD males are shorter and tend to be at the extremes of weight and BMI compared with the general male paediatric population [10].

In DMD, despite the absence of steroid use, weight gain seems to increase from ages 7–10 years, and the average weight of ambulatory steroid-naive males with DMD is greater than the averages on the CDC 2000 growth charts [11,12]. This observation suggests that weight gain in DMD is not only a side effect of steroid therapy. It has been found that the greatest risk for overweight/obesity occurs in the preteen to teenage years (9–17.7 years), while undernutrition and weight loss become prevalent at approximately 18 years of age [12,13]. In light of these data, it is recommended that patients should be routinely weighed to identify those individuals with a trend towards excess weight gain or weight loss requiring dietetic evaluation.

Regarding height, several studies have demonstrated that children with DMD are shorter, on average, than the typical male child, regardless of steroid therapy [8,10]. In DMD, height outcome may be predicted by genetics because distal deletions of the DMD gene are more frequently associated with shorter stature [8]. Central mutations are also associated with short stature, but to a lesser degree [8].

Most studies evaluating height in DMD patients have focused on the years prior to loss of ambulation. In a non-ambulatory population it is difficult to obtain reliable and comparable height measurements. Limited range of movement, joint contractures, and scoliosis are additional factors that make for less accurate height measurements. Haapala et al. evaluated the agreement between measured and estimated height in children and young adults with cerebral palsy, and demonstrated that the preferred method when standing height cannot be obtained is to take the sum of individual segmental lengths [14]. It is necessary to standardize the methods for measuring height in non-ambulating patients with DMD or other NMDs in order to disseminate standards and obtain comparable data [15].

Given the noted increases in weight and short stature, it is not surprising that BMI tends to be higher in children with DMD compared with CDC growth trends [10]. DMD patients with body weight and BMI in accordance with standard growth charts may show good nutritional status without a need for dietetic evaluation, but because lean muscle mass can be greatly diminished, they could present with excessive body fat [5,16]. Another aspect to consider is that when the measurement of height is difficult because patients cannot stand up, the calculation of BMI may be approximate, and any error in the measurement of height would lead to an exponential error in the calculation of BMI because height is squared [14].

Overall, the available data documenting an increase in weight, a decrease in stature, and an increase in BMI in patients with DMD should be confirmed in other NMDs, in which it is advisable to carefully use standard growth charts. In addition, due to the limitations reported above, the use of BMI does not seem to be the best way to evaluate the growth of children with NMDs, and body composition should always be considered when interpreting anthropometric measures in these patients.

## 3. Overnutrition

In DMD and other NMDs, patient overnutrition is multifactorial due to decreased caloric needs associated with decreased physical activity and resting energy expenditure (REE). The excess caloric intake is due to the possible use of medication resulting in increased appetite and caregivers’ compassion which causes a lack of caloric restriction (Table 1) [5]. In this group of patients, risks of obesity with ageing are insulin resistance (with increased risk of carbohydrate intolerance and diabetes), dyslipidemia, hypertension, and obstructive sleep apnea [5]. Other complications of being overweight are the acceleration of disease progression due to the exertion of extra force on already weak muscle groups, increased respiratory involvement with worsening pulmonary and cardiac function, and deterioration of skeletal malformations with increased need for orthopaedic surgery [5,17]. In addition, obesity worsens the ability of parents and caregivers to transfer and assist the patient in daily activities when patients lose their independence [15]. Furthermore, as in the general population, being overweight has adverse psychological impacts to further debilitate patients with chronic disease and physical disability [15]. Therefore, obesity can worsen the quality of life of these patients as well as their caregivers.

Chronic treatment with corticosteroids increases the risk of becoming overweight, insulin resistance, and type 2 diabetes mellitus [15]. Corticosteroids may stimulate appetite and food intake and act on liver and fat cell metabolism to promote insulin resistance, hyperglycaemia, and visceral adiposity. Glucose metabolism should be evaluated in the presence of excess weight gain with paired glucose and insulin levels, glycosylated haemoglobin levels, and the oral glucose tolerance test [15].

The mainstays of prevention and treatment of becoming overweight in patients with NMDs is dietary control, as an increase of physical activity obviously has limited practical value. Therefore, dietetic advice can be beneficial to control caloric intake and excess weight gain when implemented prior to the commencement of steroid treatment [15]. A low glycaemic index diet may be useful to control alterations of glucose metabolism, and is based on the avoidance of simple sugars, the consumption of complex carbohydrates that produce relatively small changes in blood glucose, portion control, and increased fibre consumption [15]. Examples of carbohydrate-containing foods with a low glycaemic index include dried beans and legumes, all non-starchy vegetables, most fruits, and many whole-grain breads and cereals. Furthermore, dietetic advice to limit caloric intake is useful, and recommendations include reducing the intake of sugar-containing beverages and calorically-dense foods, paying attention to meals consumed outside the home, increasing the consumption of fruits and vegetables, limiting the addition of oils and fats, and the consumption of only small portions of sweet foods for breakfast and not at the end of meals [18,19]. Finally, behaviour modification techniques include the consumption of meals with family and encouraging patients to eat slowly while recognizing satiety cues [20].

## 4. Undernutrition

The transition from overnutrition to undernutrition usually occurs with disease progression. Mehta et al. revealed a decline in both weight and BMI *Z*-scores across a three-year time period in 60 children aged 2–12 years old with spinal muscular atrophy [21]. A significant decline in BMI was noted in 47% of the patients, and the prevalence of severe malnutrition increased from 2% to 17% after a period of three years.

Decreased muscle strength is the main cause of hypoalimentation (Table 2) [22]. Dysphagia, gastrointestinal problems (i.e., constipation, delayed gastric emptying), prolonged mealtime, and dependent feeding are all consequences of muscle weakness [22]. Furthermore, the presence of respiratory failure in the late stage of the disease can cause increased energy requirements [22]. The consequence of hypoalimentation and increased energy requirements is a negative energetic balance and weight loss.

Moreover, a variety of swallowing difficulties are reported in patients with NMDs (i.e., facial weakness, reduced mastication, and poor tongue coordination) [6,12,22]. These problems result in increased mealtime with a consequent decrease in food intake, weight loss or an inability to gain weight; food inhalation into the airways with subsequent breathing problems and recurrent respiratory infections; and increased episodes of choking, coughing, and spluttering while eating resulting in embarrassment and psychological difficulties [6,12,22,23].

Undernutrition can deteriorate respiratory function and blunt immunological responses with increased risk of chest infections and a negative impact on quality of life [22]. Considering all these observations, an integrated multidisciplinary treatment appears to be mandatory to recognize signals that can indicate reduced food intake to avoid a negative impact on nutritional status.

## 5. Resting Energy Expenditure (REE) and Energy Requirement

Skeletal muscle metabolism is a major determinant of REE, and it is altered by the severe muscle loss that characterizes NMDs. Shimizu-Fujiwara et al. investigated REE in 77 DMD patients aged 10–37 years at various disease stages [13]. REE was significantly lower than the corresponding value in controls. At the advanced stage of the disease, abnormal development and atrophy of the liver—common findings in DMD—can contribute to the decrease in REE. A weak positive correlation between REE and serum levels of rapid turn-over proteins (prealbumin and cholinesterase synthesized by liver) was previously observed [13].

Hankard et al. studied REE in 13 DMD children (aged 8–13 years) and hypothesized that muscle mass loss would decrease REE and therefore contribute to the onset of obesity [24]. Alternatively, Zanardi et al. evaluated nine patients aged 6–12 years, showing that a loss of muscle mass in DMD patients was not associated with a reduction in REE [25].

It has been determined that DMD patients require fewer calories compared to healthy children (80% of the recommended caloric intake for healthy children in ambulatory boys and 70% in non-ambulatory boys) [15]. Therefore, caloric intake should be individualized based on physical activity and the capability of ambulation [15]. However, decreasing caloric intake has the potential to induce a negative energy balance, and in NMDs this condition may increase the loss of lean body mass, which, once lost, does not have the potential to regenerate [17].

Little is known about how energy requirements in NMDs differ from those in DMD. For the particular body composition of NMD patients, predictive energy formulas based on weight may have limited value. Further research is needed to understand energy requirements and to develop specific guidelines for energy prediction in NMD patients. In the interim, overweight prevention is an appropriate treatment for NMD patients; this involves dietary education in the early years after diagnosis and prior to the initiation of corticosteroid therapy. In obese patients, the dietitian should plan a programme for caloric restriction, while considering the devastating effects of excess weight and the potential for muscle loss associated with inducing a negative energy balance.

## 6. Protein and Fluid Requirement

Little literature is available about specific protein requirements in NMD populations, and it is not possible to draw conclusions. Protein intake should meet the recommended dosage for age because there is no evidence suggesting that NMD patients require additional protein intake.

There is no literature available regarding fluid intake in NMD patients; therefore, no conclusion can be drawn. However, the intake of adequate fluid is recommended to contrast an increased risk of constipation. Fluid calculations based on height and weight are not recommended, because it is often difficult to obtain a true measure of height. It is preferable to use formulas based on weight only (i.e., the Holliday–Segar method), considering individual requirements [5].

## 7. Gastrointestinal Complications

Complications of the gastrointestinal (GI) tract are relatively frequent in NMDs. Pane et al. evaluated GI involvement in 118 DMD patients with ages ranging between 3 and 35 years, and observed that GI symptoms were reported by 47% of patients [12]. The most common GI complications in NMD patients are delayed gastric emptying, gastroesophageal reflux (GER), and constipation (Table 3) [5]. With increasing survival, gastric and intestinal dilatation related to air swallowing due to ventilator use have also been reported [7].

The altered function of gastric smooth muscle cells in NMD patients causes delayed gastric emptying [5]. Borrelli et al. demonstrated that gastric emptying time in patients with DMD was significantly delayed compared with controls and was also worse at follow-up as the disease progresses [26]. Delayed gastric emptying can contribute to GER [5]. In the study by Pane et al., the majority of patients experienced occasional episodes of heartburn, but GER requiring pharmacological treatment was reported in only 4% of patients with DMD [12]. The presence of GER also increases possible risks for aspiration.

Constipation usually occurs in the second decade of life in patients with NMDs, and increases with age [5]. Pane et al. reported that 36% of patients with DMD experienced constipation, and this problem was more frequently reported after 18 years (60% of the patients) [12]. Colon smooth muscle involvement is due to immobility, weakness of abdominal wall muscles, and inadequate fluid intake [5]. These factors are responsible for slower GI transit, increased permanence of stool in the colon for water absorption, and consequent hard, dry stool. Decreased appetite is a complication of constipation, and consequently, it is very important to treat constipation early—especially in malnourished patients [5]. Dietary advice to prevent and/or treat constipation includes adequate fluid intake together with an increase of dietary fibre consumption. Fibre supplementation without satisfactory hydration might worsen symptoms and result in large amounts of hard stool.

## 8. Dysphagia

Dysphagia is common in NMD patients and affects about one third [27]. Due to the muscle weakness that characterizes all NMDs, oral motor activities are impaired [1]. Furthermore, increased risk of aspiration predisposes to respiratory complications, and this is worsened by co-existing respiratory muscle weakness in patients with compromised airway defence mechanisms and severe coughing [4,23]. Dysphagia can also lead to social and psychological consequences with worsening quality of life that may be associated with loss of satiety, fear of choking, embarrassment, and social isolation secondary to coughing, spluttering, and prolonged feeding times [23].

Table 4 summarizes the main causes of dysphagia. In most NMDs, dysphagia is mainly related to weakness of the oral muscles rather than to the incoordination of sucking, swallowing, and breathing [1]. In progressive NMDs, the majority of children have learned to chew, but masticatory problems may arise due to increasing weakness of masseter and temporal muscles in combination with weak tongue movements, resulting in prolonged mealtimes and feeling of irritation in the throat and choking [1]. Children with non-progressive NMDs (e.g., congenital myopathy) are often not able to feed in the neonatal period, and consequently need to be started on slow tube feeding with oral nutrition to avoid possible pharyngeal dysphagia [1]. In slowly progressive NMDs, feeding and swallowing problems develop insidiously, thus close professional monitoring is needed from an early stage [1].

Unintentional weight loss or decline in the expected age-related weight gain can be a sign of dysphagia [7]. Touissant et al. considered an unintentional weight loss greater than 10% in a year to be clinically significant, but it is necessary to consider the initial morphology of patients [27]. Overweight patients have adequate fat reserves, and consequently, in those with good appetite, a diet with high calorie fluids may minimize weight loss [27]. In contrast, underweight patients have limited fat reserves and consequently more intensive nutritional therapy is needed [27].

The primary aim in the treatment of feeding and swallowing disorders is to prevent choking and to avoid aspiration pneumonia [1]. Recent studies have suggested that thickening fluid is probably not appropriate in NMDs [28]. In central forms of dysphagia (i.e., cerebral palsy), poor neurological coordination of the swallowing function is often a risk factor for aspiration during fluid intake. Alternatively, in the neuromuscular form of dysphagia, progressive muscle weakness is the main characteristic, which accompanies solid rather than liquid intake [27,28]. Consequently, it is difficult for children with NMDs to manage thick liquids and solid foods as they experience more problems with post-swallow residues when consuming these substances than when consuming thin liquids. In the presence of mastication problems, softer foods and smaller pieces of food are recommended to ensure that patients have an adequate intake of nutrients (i.e., protein, iron, fibre, and calories) [1].

Poor head control is often present in advanced stages of NMDs, and contributes to worsened feeding and dysphagia [1]. Adaptation of the head posture of children improves the efficiency of swallowing in these cases [1].

Toussaint et al. presented an algorithm to facilitate clinical decisions regarding dysphagia management in patients with DMD [27]. If difficulty in swallowing is present but it occurs without weight loss, the presentation of meals may be modified to allow for easier swallowing by reducing the efforts of chewing and transporting of the bolus [27]. It is advised to stop solid food, to promote pureed meals, and to rinse the throat regularly during and after meals with an appropriate amount of fluid [27]. In the case of unintentional weight loss, a high-caloric diet is proposed (maximal calories in a minimal volume), as well as increasing the caloric and protein density of meals. If the intake of natural food is not adequate, high-energy drinks or powders should be added [27]. A similar approach should be proposed in other NMDs, but specific studies on dysphagia in the various cohorts of patients with NMDs are needed before defining appropriate algorithms for the single conditions.

## 9. Enteral Nutrition

Enteral nutrition is often required in NMD patients. It is recommended to discuss enteral nutrition with the patient and the family at an early stage of the disease progression, giving enough time for a possible better outcome [17]. Early discussion and an early decision of percutaneous endoscopic gastrostomy (PEG) can also reduce risks associated with anaesthesia if respiratory capacity is not yet compromised [17]. Toussaint et al. recommended PEG when a high caloric diet trial is unsuccessful (i.e., decreased weight even with high calories) [27]. DiVito and Meyers recommend placing a feeding tube if one of these conditions is present: the child aspirates, mealtimes are longer than 30 min, the child is unable to meet nutritional needs, there is weight loss or lack of weight gain for 3 months, there is a decrease of two or more weight or height percentiles [29]. Ramelli et al. reported that PEG was associated with a reduced frequency of chest infections and consequent hospitalization, and it appeared to be more effective in improving weight and height than the use of oral supplementation [30]. The results of Martigne et al. showed improved weight status in many patients with DMD after PEG placement, and likely increased life span and/or quality of life [31]. After PEG placement, if the patient is able to eat with no risk of aspiration, oral feeding can be maintained, but without the need to reach the entire energy requirement [27]. Alternatively, if aspiration is evident, oral feeding is prohibited and the whole volume of calories and nutrients is provided by enteral nutrition [27].

This means that a multidisciplinary team including a dietitian, a gastroenterologist, and a swallowing therapist are needed to: (a) maintain the best nutritional status to prevent both undernutrition and overnutrition; (b) manage GI problems; and (c) monitor and treat dysphagia to prevent aspiration pneumonia and weight loss (Table 5).

## 10. Reduced Bone Mass

It is not known whether NMDs directly affect bone, but the necessity of long-term therapy with corticosteroids exposes the patients to unwanted side effects, such as loss of bone mass and an increased risk of fractures [3]. Other risk factors for poor bone health in NMDs include reduced weight-bearing activity and muscle weakness, with a consequent risk of fractures, osteopenia, osteoporosis, scoliosis, bone pain, and poor quality of life [7]. In the study of Bianchi et al., in about two-thirds of DMD cases, calcifediol supplementation, adjustment of dietary calcium intake to the recommended dose, and reduced sodium intake to avoid calciuric effects were able to reduce bone resorption, correct vitamin D deficiency, and increase bone mass [3].

Reaching the recommended calcium intake during childhood and adolescence is necessary to achieve optimal peak bone mass. A calcium-rich diet based on dairy products is usually appreciated by children, and may have the greatest benefit on bone accrual, while calcium supplements are sometimes not well tolerated [3]. Such a diet can become a healthy habit for all patients at risk of low bone mass. The problem of increased fracture risk will become more serious with the prolonged survival of patients.

## 11. Conclusions

Nutritional complications are very frequent in DMD, but they are sometimes underestimated. However, data collected from DMD patients should be extended to the other NMDs. Studies on the prevalence of overnutrition and undernutrition, GI complications, infectious diseases, dysphagia, and reduced bone mass in all the different types of NMDs are urgently needed. Additionally, appropriate percentiles of weight, height, BMI, and body composition appear to be extremely important to improve a patient’s NMD management. Furthermore, problems of drug side-effects on growth and quality of life must be taken in consideration. Based on different muscle involvement and degree of impairment, complications might be different among NMDs. Moreover, gut microbiota could also influence processes including homeostasis, drug pharmacokinetics, and therapeutic response in NMD patients. Meanwhile, appropriate patient management should include dietetic assessment at diagnosis before initiating corticosteroids: (1) when patient is underweight; (2) unintentional weight loss or poor weight gain; (3) patient is overweight or at risk of becoming overweight; (4) major surgery is planned; (5) patient is chronically constipated; or (6) dysphagia is present. In addition, due to the longer life expectancy in NMD patients, nutritional issues and complications related to adult age should be considered. Specifically, increased weight gain together with the inability to exercise can probably increase the risk of developing a cluster of cardiovascular risk factors, as well as metabolic syndrome. Further research on this new adult population with NMDs will enable improved quality of life due to the avoidance of nutritional challenges.

## Figures and Tables

**Table 1:** Overnutrition in patients with neuromuscular diseases (NMDs).

Risk Factor
Decreased caloric needs
Decreased resting energy expenditure
Decreased physical activity
Excessive caloric intake due to increased appetite because of medication
Lack of caloric restriction by parents

**Table 2:** Undernutrition in patients with neuromuscular diseases (NMDs).

Main Causes
Decreased muscle strength
Dysphagia
Gastrointestinal problems (i.e., constipation, delayed gastric emptying)
Prolonged mealtime
Dependent feeding
Increased energy requirements because of respiratory failure
Swallowing difficulties

**Table 3:** Gastrointestinal complications in patients with neuromuscular diseases (NMDs).

Gastrointestinal Complication	Pathogenesis
Delayed gastric emptying	Altered function of gastric smooth muscle cells
Gastroesophageal reflux	Delayed gastric emptying
Constipation	Immobility
	Weakness of abdominal wall muscles
	Inadequate fluid intake

**Table 4:** Causes of dysphagia in patients with neuromuscular diseases (NMDs).

Main Causes
Weakness of the oral muscles
Incoordination of sucking and swallowing
Difficulties in breathing

**Table 5:** Main recommendations for the management of nutritional complications in patients with Duchenne muscular dystrophy (DMD).

Main Causes
Identification of a multidisciplinary team including a dietitian, a gastroenterologist, and a swallowing therapist
Maintenance of the best nutritional status to prevent both undernutrition and overnutrition
Management of gastrointestinal problems
Monitoring and treatment of dysphagia to prevent aspiration pneumonia and weight loss
